# Post-pandemic upsurge in Group A *Streptococcus* infections at an Italian tertiary university hospital

**DOI:** 10.1128/spectrum.02494-24

**Published:** 2025-03-26

**Authors:** Gabriele Arcari, Federica Novazzi, Lorenzo Colombini, Francesca Drago Ferrante, Sara Boutahar, Angelo Paolo Genoni, Gianluca Cassani, Paolo Gigante, Mattia Carbotti, Alessandro Bianco, Mariana Tirziu, Riccardo Capuano, Renee Pasciuta, Francesco Iannelli, Nicola Clementi, Francesco Santoro, Nicasio Mancini

**Affiliations:** 1 Department of Medicine and Technological Innovation, University of Insubria 19045 , Varese, Italy; 2 Laboratory of Medical Microbiology and Virology, Ospedale di Circolo e Fondazione Macchi 18709 , Varese, Italy; 3 Department of Medical Biotechnologies, University of Siena 574285 , Siena, Italy; 4 Laboratory of Medical Microbiology and Virology, Vita-Salute San Raffaele University 18985 , Milan, Italy; 5 IRCCS San Raffaele Hospital 574703 , Milan, Italy; Department of Molecular and Translational Medicine, University of Brescia, Brescia, Italy

**Keywords:** *Streptococcus pyogenes*, invasive infections, *emm* typing, genomic epidemiology, phylogenetics, antimicrobial resistance

## Abstract

*Streptococcus pyogenes* (Group A *Streptococcus*; GAS) is a pathogen of global significance. In the pre-antibiotic era, GAS was a major cause of childhood morbidity and mortality, but its spread rapidly declined until the mid-2010s. The continuing increase in GAS infections, associated with the expansion of the M1_UK_ lineage, was observed first in the United Kingdom (UK) and, later, globally. Here, we endeavor to assess the various determinants underlying the post-pandemic GAS upsurge, with a focus on microbial genomic features. We performed an epidemiological analysis of all laboratory-confirmed GAS infections identified between June 2018 and June 2024 at a tertiary University Hospital located in Northern Italy, dividing them into three levels of severity: mild, moderate, and invasive GAS infections. A subset of 34 representative GAS isolates identified in the post-pandemic period were subjected to short- and long-read whole genome sequencing (WGS). Of the 531 GAS cases analyzed during this period, the majority (415, 78.2%) occurred in the last two years. This increase in GAS cases correlated with a significant shift in infection severity: among the 118 GAS cases identified in the June 2018–May 2022 period, only one resulted in an invasive infection (1/118, 0.8%). In contrast, among the 531 GAS cases identified in the June 2022–May 2024 period, 32 caused invasive infections (32/531, 7.9%). WGS of 34 isolates (including 15 invasive isolates) identified 11 different *emm* types, the most frequent being *emm*1 (9 isolates) followed by *emm*12 (7 isolates), then *emm*89 and *emm*28 (4 isolates each). Among the *emm*1 isolates, the M1_UK_ sublineage was the most represented (8 out of 9 isolates), with the remaining “singleton” belonging to the M1_13SNP_ sublineage.

**IMPORTANCE:**

*Streptococcus pyogenes* (GAS) is a narrow-spectrum pathogen, circulating only in humans. Following the loosening of various public health measures implemented to face the challenge of the COVID-19 pandemic, a significant rise in GAS cases has been observed. Our study revealed a significant rise in GAS cases, particularly invasive infections, over the last two years. Genomic analysis identified multiple sequence types, including isolates belonging to an emerging lineage named M1_UK_. These findings underscore the importance of ongoing surveillance and genomic monitoring of GAS infections, especially considering their rising incidence and severity. Public health strategies should consider not only microbe-associated aspects but also host-associated and external factors to effectively address this resurgence and prevent future outbreaks.

## INTRODUCTION

*Streptococcus pyogenes* (Group A *Streptococcus*; GAS) is a narrow-spectrum pathogen, which only circulates in humans, and whose transmission almost exclusively occurs from person to person, i.e., respiratory droplets or direct contact (1). GAS is a clinically relevant microorganism causing, in the main, non-invasive infections, e.g., pharyngitis and impetigo, but it is also responsible for life-threatening invasive infections, e.g., sepsis and meningitis (1).

Starting from 2014, an increase in GAS infections (in the form of scarlet fever and invasive infections) was observed in the United Kingdom (UK), mostly linked to the spread of a serotype M1 virulent lineage, which was characterized by an enhanced expression of the scarlet fever toxin SpeA (2): the so-called M1_UK_ lineage (3). After a reduction in the first two years of the COVID-19 pandemic, various European countries (Denmark, Ireland, France, Germany, the Netherlands, Spain, and Sweden) have reported a significant increase in GAS cases (4–8).

Non-pharmaceutical interventions (NPIs) and societal behavioral changes due to the COVID-19 pandemic caused a reduced exposure to endemic viruses, which then resulted in a wider diffusion of viral-mediated respiratory transmittable diseases after the relaxation of NPIs (9). It is not clear whether this also applies to non-viral pathogens.

Besides sporadic events occurring between June 2020 and March 2022, definitive NPI easing in the Italian region of Lombardia gradually started in the Spring of 2022. Recent GAS molecular epidemiology analyses have highlighted how the M1_UK_ lineage has been identified in multiple countries (7, 10–12), in some cases displacing the endemic lineages (13–15). The steep increase in GAS infections reported across Europe was also observed in our center, a tertiary University Hospital located in Northern Italy and serving as a microbiological hub for almost 500,000 people. This study is based on a comprehensive collection of clinical microbiology data leveraging state-of-the-art genomics, which aims to evaluate the factors underlying the recent GAS upsurge.

## MATERIALS AND METHODS

### Study setting and population

All medical reports documenting the identification of *S. pyogenes* at the University Hospital of Varese (Northern Italy) from June 2018 to June 2024 were included. The Laboratory of Medical Microbiology and Virology serves a population of approximately 500,000 people in mixed urban and rural settings and analyzes samples from both hospitalized and community patients Dataset S1. The laboratory retrospectively retrieved microbiological data from their records. This study did not involve the collection of detailed clinical data such as host status, comorbidities, therapies, and outcomes.

In contrast with the current dichotomous division of infections caused by *S. pyogenes* between non-invasive and invasive infections (non-iGAS and iGAS, respectively) (16, 17), we maintained the iGAS definition to illustrate severe infections but split non-iGAS into two groups, hence defining three severity levels based on the sampling source:

- mild (ear, genital, and oropharynx)
- moderate (abscesses, eye, pus, respiratory tract samples, skin, urine, and wounds), and
- iGAS (blood, cerebrospinal fluid, and other sterile fluids).

Samples yielding GAS from any sterile or non-sterile site were included. Isolates were excluded if GAS was recovered within three months of a previous isolate from the same patient, regardless of sample type.

### Bacterial identification and storage

The identification of bacterial isolates was performed by MALDI-ToF MS (VITEK-MS, bioMérieux). Following the upsurge of GAS cases reported in late 2022 in the literature, the rate of GAS detection was closely monitored in our center, including the storage of invasive strains at −80°C for further analyses.

Out of the 128 GAS isolates sampled between May 2023 and January 2024, 35 isolates were subjected to whole genome sequencing (WGS) using both short- and long-read technologies. Due to contamination with other bacterial species, one isolate (#25) was excluded from further analyses causing a numbering gap.

These 34 isolates included 15 iGAS isolates (14 from bloodstream infections and one from cerebrospinal fluid) and a convenience sample of 8 moderate and 11 mild GAS infections. Sequenced isolates included a previously known family cluster of 4 GAS (18).

### Genomic DNA purification and sequencing

Bacteria were grown until the late exponential phase (OD_590_ of 1) and high-molecular-weight genomic DNA was purified using a raffinose-based method (19). DNA was quantified using the Qubit dsDNA BR Assay Kit (Thermo Fisher Scientific).

WGS was performed using both Illumina and Nanopore sequencing technologies. Illumina was performed using the NextSeq 500/550 v2.5 Kit (300 cycles) on a NextSeq 550 device (Illumina, San Diego, CA, USA, 2 × 150 bp paired-end sequencing) (18). Nanopore sequencing was carried out as already described (20), and DNA was size selected with 0.5 volumes of AMPure XP beads (Beckman Coulter, Milano, Italy). Libraries were constructed with the SQK-RBK 114.96 kit (Oxford Nanopore Technologies, Oxford, UK) starting from 200 ng of DNA. Sequencing was performed on a GridION X5 device (Oxford Nanopore Technologies) using an R10.4.1 flow cell (FLO-MIN114). Real-time base calling was performed with Guppy high accuracy mode v7.1.4 (Oxford Nanopore Technology), filtering out reads with a quality cut-off of <Q9.

### Genome assembly and analysis

Illumina reads were analyzed using FastQC v0.11.5 and trimmed with Trimmomatic v0.39 as previously reported (18). Nanopore reads were analyzed with NanoPlot v1.42.0 (https://github.com/wdecoster/NanoPlot), then filtered to obtain 30× coverage taking 1.8 Mb as the genome size estimate using Filtlong v0.2.1 software (https://github.com/rrwick/Filtlong) with parameter “--target_bases” and assembled using Flye v2.9.3-b1797 (https://github.com/mikolmogorov/Flye). The resulting circular contig was polished with Medaka v1.11.3 (https://github.com/Nanoporetech/medaka) using the filtered Nanopore reads, followed by two polishing rounds with Pilon v1.24 (https://github.com/broadinstitute/pilon) using the trimmed Illumina reads. Assembly completeness was assessed with Bandage v0.8.1 (https://github.com/rrwick/Bandage), whereas assembly quality was evaluated with both Ideel (https://github.com/mw55309/ideel) and CheckM v1.1.3 (https://github.com/Ecogenomics/CheckM). Assembled genomes were automatically annotated with the NCBI Prokaryotic Genome Annotation Pipeline (PGAP) v6.7 (21).

Mobilome analysis was carried out as reported (20): the presence of integrative and conjugative elements (ICEs) and of integrative and mobilizable elements (IMEs) in the genomes was investigated with ICEfinder (https://bioinfo-mml.sjtu.edu.cn/ICEfinder/ICEfinder.html, accessed May 2024), whereas the presence of prophages was investigated with PHASTER (http://phaster.ca, accessed May 2024). Default parameters were used for all software unless otherwise specified. The presence of acquired antimicrobial resistance genes was detected by ResFinder (22), while amino acid substitutions associated with fluoroquinolone resistance in the GyrA and ParC proteins were analyzed by a Clustal Ω alignment with the respective sequences from reference strain SF370 (GenBank accession number AE004092).

Genetic relatedness of the isolates was evaluated with the PopPUNK tool v2.6.5 using the “--fit-model lineage” parameter for data fitting (https://github.com/johnlees/PopPUNK) as described (23). The complete genome of *S. pyogenes* strain SF370 (GenBank accession number AE004092) was used as an out-group for the phylogenetic tree construction. The WGS data for this study have been deposited in the National Center for Biotechnology Information under BioProject no. PRJNA1070447.

### Statistical analyses and epidemiological distribution

Contingency tables and χ² tests were performed using JASP (Version 0.19.0, https://jasp-stats.org/) and RStudio (Version 2024.4.2.764, http://www.posit.co/) by means of the packages: ggstatsplot (24), ggplot2 (25), and tydiverse (26). The epidemiological curve was designed in RStudio using the incidence (https://github.com/reconhub/incidence) package, binning together all GAS cases identified in the same month. The balloon plot representing the distribution of cases in different months, according to their severity, was designed using the ggballoonplot function from the ggpubr package (https://rpkgs.datanovia.com/ggpubr). A bubble chart representing GAS cases identified between May 2023 and January 2024 was created using RawGraphs 2.0 (https://app.rawgraphs.io).

Additionally, Welch’s *t*-test was used to compare the mean age between two groups (females and males), and Welch’s one-way ANOVA test was used to compare the mean age among the three severity layers (mild, moderate, and iGAS), while possible combinations of group differences were compared using the Games–Howell post hoc test; the χ² test was used to analyze the distribution of categorical variables. Results were considered significant when the *P* value was  ≤  0.05. All images were manipulated using the open-source InkScape software (https://www.inkscape.org) v1.3.2.

## RESULTS

### A six-year epidemiological tracking of GAS infections

Between June 2018 and June 2024, a total of 531 cases of laboratory-confirmed GAS infections were recorded from 510 patients at the Laboratory of Medical Microbiology of the University Hospital of Varese, North-West Italy.

**Fig 1:**
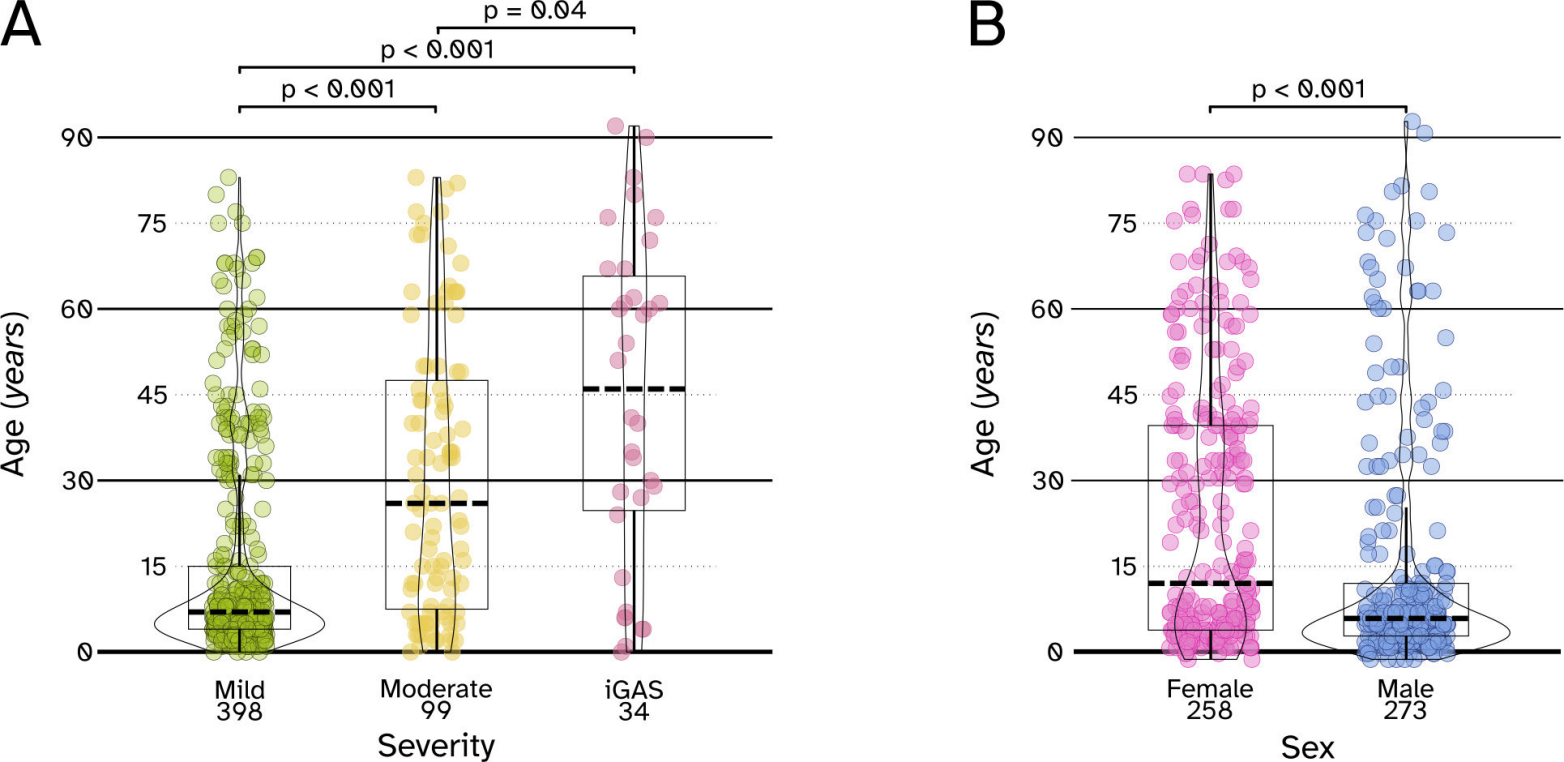
Age distribution of GAS cases according to infection severity and sex. (A) Violin plots describing the age distribution of GAS cases, split according to their severity (as defined in the Methods section). Welch’s one-way ANOVA was used to compare the mean age among the three severity groups, and the Games–Howell post hoc test was used to compare the possible combinations of group differences. Results considered significant (*P* value ≤  0.05) are reported. In the boxes within violin plots, bold dotted lines indicate median age, while upper and lower boundaries indicate 75th percentile (third quartile) and 25th percentile (first quartile), respectively. (B) Violin plots describing the age distribution of GAS cases, split according to sex. Welch’s *t*-test was used to compare the mean age between the two groups (females and males). Results considered significant (*P* value ≤  0.05) are reported. In the boxes within violin plots, bold dotted lines indicate median age, while upper and lower boundaries indicate 75th percentile (third quartile) and 25th percentile (first quartile), respectively.

A total of 398 GAS isolates were defined as “mild” (sampled from oropharynx, genital, or ear samples, 75%), 99 GAS isolates were defined as “moderate” (sampled from abscesses/pus, respiratory tract samples, skin, urine, and wounds, 18.6%), and 34 isolates were defined as “iGAS” (sampled from blood and cerebrospinal fluid, 6.4%). A significant association between infection severity and age was identified, with GAS isolated from older patients more often associated with severe infections (Welch’s one-way ANOVA *P* < 0.001, Games–Howell *P* for mild-moderate and mild-iGAS pairs < 0.001, Games–Howell *P* between the moderate-iGAS pair 0.04, Fig. 1A).

During the period analyzed, in three patients, a mild infection was followed by a moderate infection within a three-month timeframe. In all the cases, GAS was initially isolated from the oropharynx, and it was later detected either in the respiratory tract (two cases) or on the skin (one case) (Dataset S1).

The 510 patients were almost equally divided between females (249, 48.9%) and males (261, 51.1%). While female patients were significantly older than males (mean age 24.3 vs 15.1 years, *P* < 0.0001, Fig. 1B), no substantial difference in infection severity between sexes was observed.

**Fig 2:**
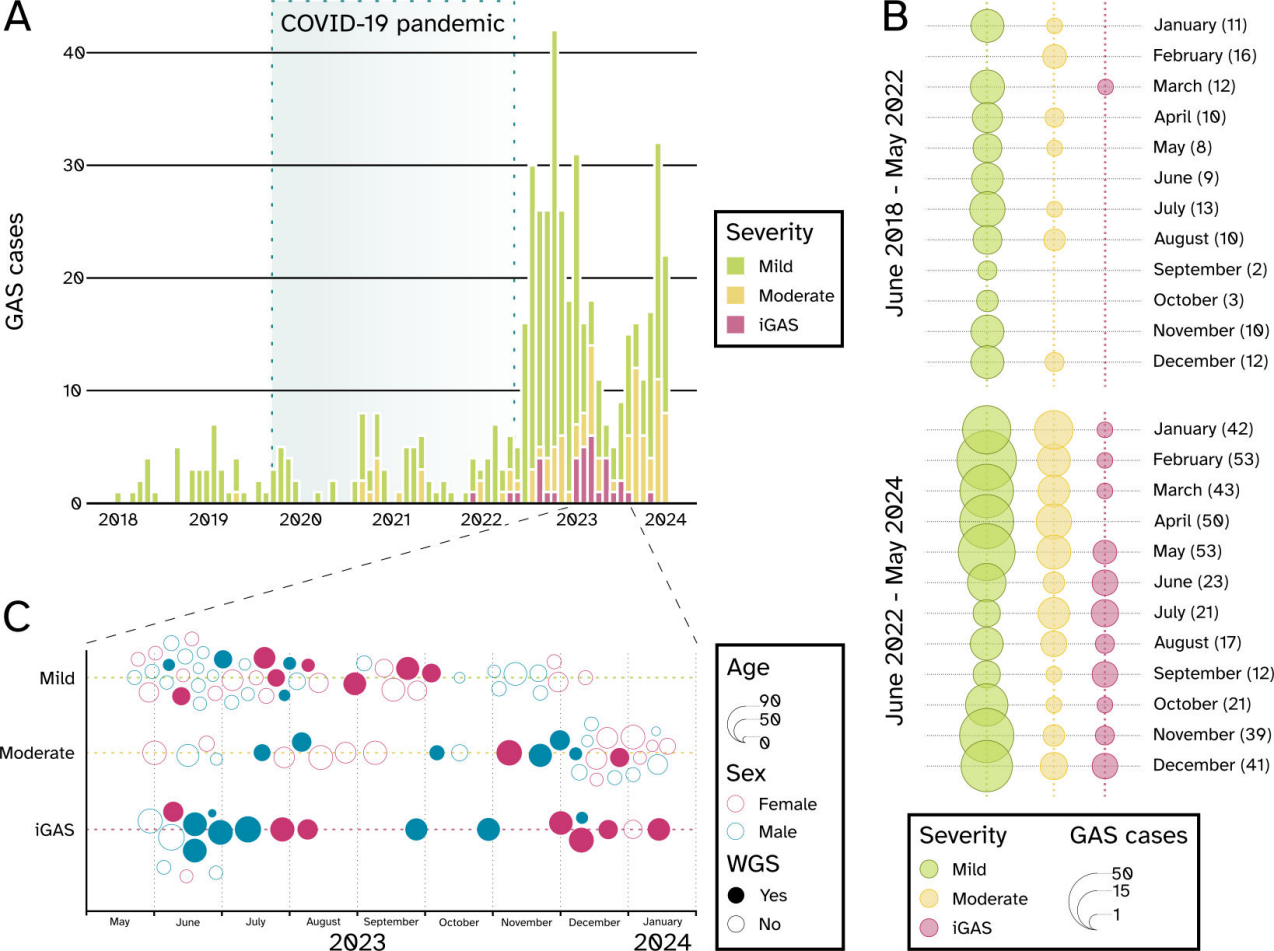
Distribution of laboratory-confirmed GAS infections. (A) Epidemiological distribution of GAS cases from June 2018 to June 2024. The severity of GAS infections (as defined in the Results section) is color-coded as follows: mild: green; moderate: yellow; iGAS: pink. The period during which COVID-19-associated restrictions were in place (from March 2020 to March 2022) is shaded in light blue. (B) Balloon plots showing the cumulative distribution of GAS cases by month of isolation split into two periods: June 2018–May 2022 and June 2022–May 2024. The severity of the cases is color-coded as follows: mild: green; moderate: yellow; iGAS: pink. The size of each balloon is proportional to the number of GAS cases in the corresponding month as per the legend. A clear seasonality was not observed either before or after the pandemic. (C) Bubble chart displaying the distribution of GAS cases, split according to severity, from May 2023 to January 2024. The chart highlights age (proportional to the size of the bubble) and sex of patients (female: dark pink; male: blue). Isolates subjected to WGS are represented by solid bubbles.

GAS cases were not uniformly distributed over the six-year period. Between mid-2018 and mid-2022, a timeframe spanning both the onset and the peak of the COVID-19 pandemic, only 116 *S*. *pyogenes* cases (21.8%) were identified at the University Hospital of Varese. However, starting from June 2022 to May 2024, a significant rise was observed with 415 cases (78.2%) recorded in just two years (Fig. 2A).

Besides the increased number of cases, a significant difference was observed in their severity: of the 116 GAS infections reported until mid-2022, 101 (87.1%) were categorized as mild, 14 (12.1%) as moderate, and only one (0.8%) as iGAS (in early 2022). On the other hand, among the 415 cases observed later, 297 cases (71.6%) were categorized as mild, 85 cases (20.5%) as moderate, and 33 (7.9%) as iGAS (χ^2^; *P* = 0.001).

This longitudinal analysis also highlights two seasonal peaks, illustrating distinct epidemiological trends. Most cases were mild and occurred between winter and spring during both the 2018–2022 and 2022–2024 periods (Fig. 2B). GAS cases declined each summer in both periods, but moderate and invasive infections had a significant relative increase during 2022–2024. Specifically, in this period, moderate cases accounted for 42.8% and 29.4% of isolations in July and August, respectively, while iGAS cases accounted for almost half of all isolations in September (41.7%, Dataset S1).

### Epidemiology of different *emm* types and analysis of the M1 sublineages

To analyze the characteristics of GAS isolates during the “post-pandemic” upsurge, 34 out of the 128 *S*. *pyogenes* isolates identified between May 2023 and January 2024 were sequenced using both short- and long-read techniques. Most of the sequenced isolates were from iGAS infections (15, 44%), while a smaller proportion was from mild (11, 32.3%) or moderate cases (8, 23.5%). Among the iGAS isolates, 14 were obtained from bloodstream infections, and one was from cerebrospinal fluid. GAS strains causing moderate (eight isolates) and mild (11 isolates) infections were selected using a convenience sampling approach. Four isolates were part of a previously identified and described family cluster (18).

The size of GAS genomes was fairly conserved with an average length of 1,840 kb (SD 61.1 kb) and did not vary according to isolation source or severity. A total of 11 different *emm* types were identified in the 34 sequenced genomes. The most represented STs (ST28, ST36, ST101, and ST52) were unambiguously correlated with a single *emm* type (Table 1). Specifically, the nine ST28 genomes carried *emm1*, the five ST36 genomes carried *emm12*, and the four ST52 and ST101 genomes carried *emm28* and *emm89*, respectively.

**TABLE 1:** Clinical attributes and genomic features of the GAS isolates subjected to WGS

ID	GenBank accession number	Age	Sex	Source	Severity	Ward	ST	*Emm* type	M1 sublineage
1	CP167025	19–65	M	Pus	Moderate	Otolaryngology	ST101	89	NA^*b*^
2	CP167024	>65	F	Blood	iGAS	ER	ST28	1	M1_UK_
3	CP167023	0–5	M	Oropharynx	Mild	Pediatrics	ST315	3.93	NA
4	CP167022	6–18	M	Oropharynx	Mild	Pediatrics	ST101	89	NA
5	CP167021	19–65	M	Pus	Moderate	Otolaryngology	ST39	4.19	NA
6	CP167020	19–65	F	Blood	iGAS	ER	ST49	75	NA
7	CP167019	19–65	F	Oropharynx	Mild	Outpatient	ST242	12	NA
8	CP167018	6–18	F	Oropharynx	Mild	Pediatrics	ST39	4	NA
9	CP167017	>65	F	Genital	Mild	Outpatient	ST28	1	M1_UK_
10	CP167016	19–65	F	Genital	Mild	Outpatient	ST49	75	NA
11	CP167015	>65	F	Wound	Moderate	ER	ST52	28	NA
12	CP167014	19–65	F	Oropharynx	Mild	Otolaryngology	ST101	89	NA
13	CP167124	19–65	M	Blood	iGAS	ER	ST28	1	M1_UK_
14^*a*^	CP167067	0–5	M	Blood	iGAS	Neonatal ICU	ST36	12	NA
15^*a*^	CP167067	19–65	F	Genital	Mild	Neonatal ICU	ST36	12	NA
16	CP167013	>65	M	Blood	iGAS	ER	ST101	89	NA
17	CP167012	19–65	F	Blood	iGAS	Gynecology	ST52	28	NA
18	CP167011	>65	M	Blood	iGAS	ER	ST403	11	NA
19^*a*^	CP167067	0–5	M	Oropharynx	Mild	Neonatal ICU	ST36	12	NA
20	CP167010	>65	M	Blood	iGAS	ER	ST28	1	M1_UK_
21^*a*^	CP167067	19–65	M	Oropharynx	Mild	Neonatal ICU	ST36	12	NA
22	CP167009	19–65	F	Oropharynx	Mild	Outpatient	ST52	28	NA
23	CP167008	>65	M	Blood	iGAS	ER	ST52	28	NA
24	CP167007	19–65	M	Blood	iGAS	ER	ST28	1	M1_UK_
26	CP167006	6–18	M	Wound	Moderate	Pediatrics	ST36	12	NA
27	CP167005	19–65	M	Wound	Moderate	Outpatient	ST403	11	NA
28	CP167004	0–5	M	CSF	iGAS	ER	ST382	6.4	NA
29	CP167003	>65	F	Blood	iGAS	Otolaryngology	ST28	1	M1_UK_
30	CP167002	19–65	F	Blood	iGAS	ER	ST89	94.1	NA
31	CP167001	19–65	M	Pus	Moderate	Surgery	ST28	1	M1_UK_
32	CP167123	6–18	M	Wound	Moderate	ER	ST28	1	M1_UK_
33	CP167000	19–65	F	Blood	iGAS	ER	ST28	1	M1_13SNPs_
34	CP166999	19–65	F	Pus	Moderate	Otolaryngology	ST46	22	NA
35	CP166998	19–65	F	Blood	iGAS	ER	ST36	12	NA

^*a*^ Isolates part of a documented family outbreak collected from related individuals residing in the same household (18). Samples 15 and 21 were obtained from adults at NICU as part of the epidemiological investigation.

^*b*^ NA, not applicable, since these isolates do not belong to the M1 lineage and sublineage cannot be assessed.

Of the 15 iGAS isolates sequenced, 6 belonged to ST28 (*emm1*), 2 to ST36 (*emm12*), and 2 to ST52 (*emm28*), while the others were singletons. However, even though a substantial quota of iGAS was typed as ST28 (6/15, 40%) and, conversely, a substantial quota of ST28 isolates were the cause of iGAS infections (6/9, 66.7%), no significant difference was observed when evaluating the severity of the isolation source relative to the *emm* type. This observation was further corroborated by a complementary analysis using the presence of *emm1* as a dichotomous variable.

Patients infected with *emm1* GAS, however, were significantly older than those infected with other lineages (mean age for *emm1* 57.3 years, SD 22.6 years; mean age for non-*emm1* 36.2 years, SD 26.1 years; Mann-Whitney *t*-test *P* = 0.016). Most of the *emm1* isolates identified and sequenced in this study displayed 27 conserved SNPs in specific sites that distinguish the emerging M1_UK_ lineage from the M1_Global_ lineage, except for isolate #33 (accession number CP167000) which belonged to the M1_13SNPs_ sublineage (27). However, when exploring the SNPs defining the M1 sublineages relative to the *emm1* reference strain MGAS5005 genome (accession number CP000017), an additional SNP was identified in the transcriptional regulator *rofA* gene. Specifically, a synonymous substitution (C650T) was observed in all nine ST28 e*mm1* genomes in this study, including isolate #33.

### Phylogenetic structure of the “post-pandemic” GAS isolates

**Fig 3:**
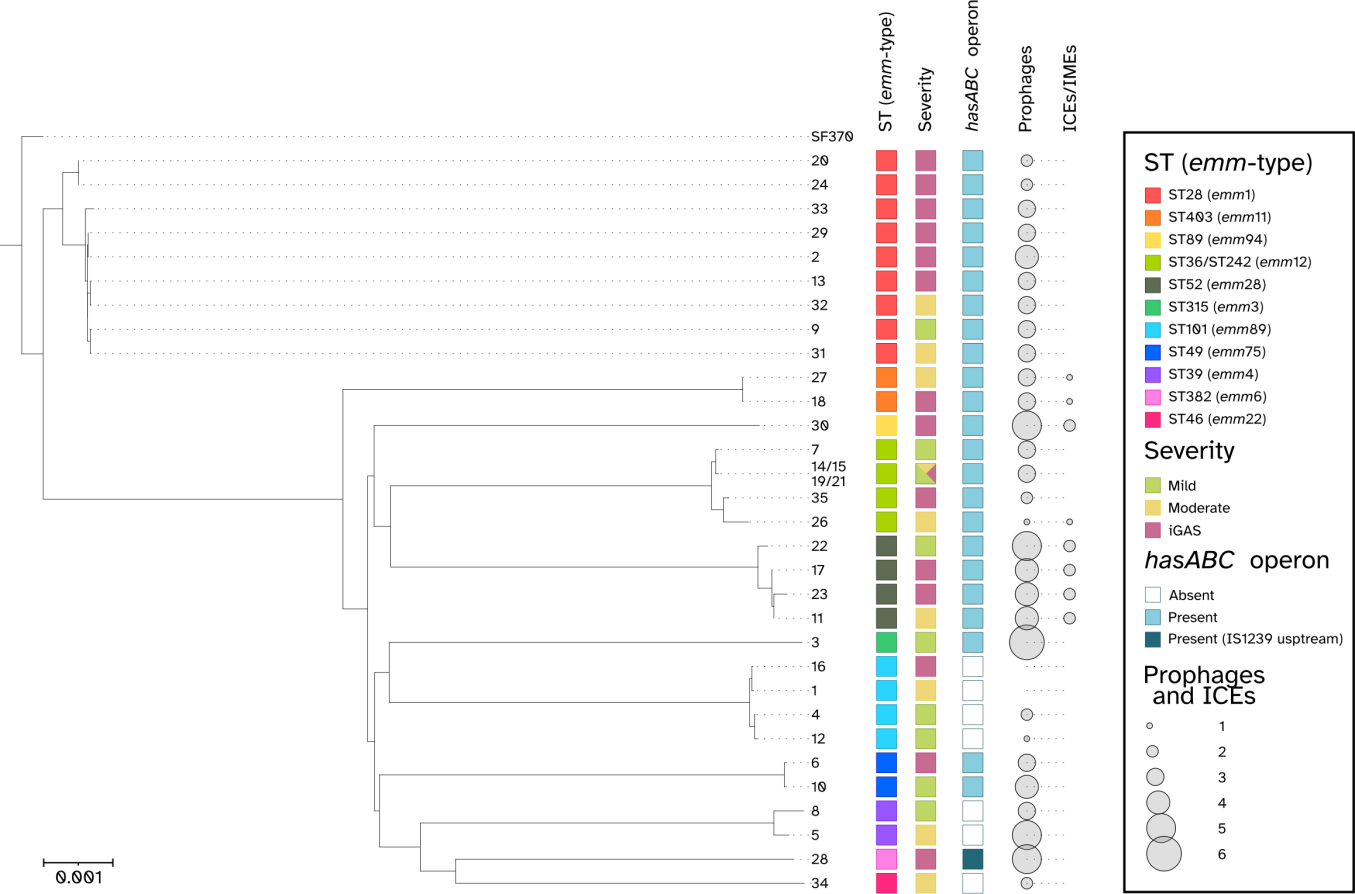
Annotation- and alignment-free phylogenetic analysis based on K-mers performed on 35 GAS isolates, 34 were subjected to WGS in this study and the reference M1 GAS isolate SF370 (accession number GCA_000006785.2). Starting from the left, metadata report the sequence type (ST) and the associated *emm* type (color-coded according to the legend), the severity of GAS infections (as defined in the Results section, color-coded as follows: mild: green; moderate: yellow; iGAS: pink), the presence of the *hasABC* operon (color-coded as follows: empty square: absent; light blue square: present; dark blue square: presence of the *hasABC* operon flanked by an IS*1239*), and the number of prophages and integrative and conjugative elements (ICEs)/integrative and mobilizable elements (IMEs) proportional to the size of the gray circles.

The 34 sequenced GAS isolates, which included 11 causing ‘mild’ infections, 8 causing ‘moderate’ infections, and 15 causing iGAS infections (Table 1), were phylogenetically analyzed using PopPUNK, revealing significant genetic diversity (Fig. 3). The genomes clustered into two main branches: one more conserved encompassing all ST28/*emm1* isolates (including the genome of the reference M1 GAS isolate SF370, accession number AE004092) and the other more heterogeneous, including all remaining isolates.

The nine ST28 genomes in this collection displayed varying degrees of conservation within their core genome. A cluster of seven genomes were highly similar, with pairwise differences ranging from 0 to 80 SNPs (Dataset S2). In contrast, two ST28 genomes, #20 and #24, accession numbers CP167010 and CP167007, respectively, both from isolates causing iGAS infections, were more divergent, differing from the cluster by 1,180–1,193 SNPs. The 25 non-ST28/*emm1* isolates, alternatively, were clustered into 10 ST-specific branches. Among these, four ST36/*emm12* isolates (#14, #15, #19, and #21), which were part of a previously described family outbreak (18), showed no differences in their genomic sequences and were collapsed into a single leaf in the phylogenetic tree, linked to a single accession number (CP167067).

### Genomic features of the “post-pandemic” GAS: capsule operon, mobile genetic elements, and antimicrobial-resistant genes

Among the 34 sequenced GAS isolates, the presence of the *hasABC* operon-encoded capsule biosynthesis cluster was found in most isolates (27/34, 79.4%) with three lineage-associated exceptions: the already described unencapsulated ST101/*emm89* (28), ST39/*emm4* (29)*,* and ST46/*emm22* (30) clones. However, in addition to capsulated and unencapsulated “post-pandemic” GAS, one isolate in our study (isolate #28, accession number CP167004, causing a cerebrospinal fluid infection) displayed a highly mucoid phenotype. A genomic inspection of the capsule operon highlighted the presence of the Insertion Sequence IS*1239* upstream of the *hasABC* operon, oriented in the opposite direction.

A total of 39 different prophages, 3 integrative conjugative elements (ICEs), and 3 integrative mobilizable elements (IMEs) were identified in the 34 analyzed genomes, with no specific correlation to either antimicrobial susceptibility patterns or infection severity (Fig. 3; Dataset S3). Among the 39 prophages identified, 23 were previously characterized, including 4 satellite prophages, while 16 were novel prophages with less than 90% nucleotide homology to known prophages. ICEs and IMEs were either previously uncharacterized or represented novel composite structures of previously described elements (Dataset S3). Isolates #1 and #16 did not contain any prophages or ICEs/IMEs, whereas the other isolates carried between 1 and 6 prophages, with eight isolates also harboring 1 or 2 ICEs/IMEs (#11, #17, #18, #22, #23, #26, #27, #30). Isolate #30 (accession number CP167002) carries a 60.9 kb long composite ICE (ICE*GAS30.1*) with a backbone homologous to *S. pyogenes* ICE*Sp*1108 (GenBank accession number FR691054, 31) containing the erythromycin resistance gene *erm*(TR), a variant of *erm*(A). A Tn*916*-like element, carrying the *tet*(M) tetracycline resistance gene, is inserted upstream of *erm*(TR) and replaces the IS*110* family gene of ICE*Sp*1108. (Table S1).

The ST403 isolates #18 and #27 (accession numbers CP167011 and CP167005, respectively) carried another novel 65.2 kb long composite ICE, namely ICE*GAS18.1*, containing the *S. pneumoniae* transposon Tn*6003* (GenBank no. AM410044.5 (32), carrying four resistance determinants (*tet*(M), two copies of the *erm*(B) gene, and *aph(3')-III*) integrated into the backbone of a larger ICE homologous to *Streptococcus anginosus* ICE*San49.2* (PP062800.1).

Finally, isolate #26 (accession number CP167006) contained a 63.7 kb long composite ICE carrying a Tn*916-*like element bearing both *tet*(M) and *erm*(B) resistance determinants embedded into a novel ICE backbone (Table S1).

Two isolates (#28 and #30) were resistant to quinolones, despite lacking acquired resistance genes. Targeted analysis revealed mutations in the *parC* gene, with substitutions at Ser79 (to Ala in #28 and Phe in #30). Isolate #30 also had an additional mutation (Ala542Thr) in *parC* and unique substitutions in *gyrA* (Gly252Ser and His382Ser).

## DISCUSSION

Starting from 2015, a significant shift in GAS infection pattern and severity has been noted globally. Several studies highlighted the contribution of the M1_UK_ lineage (12, 33), which is characterized by heightened expression of streptococcal pyrogenic exotoxin A (SpeA) (3, 10).

From mid-2022, Italy also observed this GAS re-emergence (34, 35). This six-year epidemiological tracking, conducted between June 2018 and June 2024, noted a total of 531 GAS cases, with 398/531 (75%) classified as mild, 99/531 (18.6%) classified as moderate, and 34/531 (6.4%) classified as iGAS. The temporal distribution of GAS infections displayed a marked post-pandemic increase, coinciding with a rise in moderate cases and with the advent of iGAS cases. In this study, we observed how the surge of iGAS cases that started in 2022 is only partially attributable to *emm1*, suggesting that this “post-pandemic” upsurge could also be correlated with a somehow higher susceptibility of the host, as for other pathogens (36).

Short- and long-read WGS was performed on 34 isolates and, albeit limited by geographical, temporal, and numerical constraints, emphasizes how a higher proportion of *emm1* was observed among iGAS cases, with the M1_UK_ lineage causing 5/15 (33.3%) of iGAS infections.

As a complement, data from the six-year epidemiological distribution of GAS provided insights about seasonal trends, with peaks in pharyngitis typically occurring in winter (37) and iGAS occurring more frequently in warmer periods (1, 38, 39) and underscores age as a relevant factor for the increase of GAS infection severity.

In terms of antimicrobial resistance, *S. pyogenes* isolates still display full susceptibility to beta-lactams, but a varying share of strains has acquired resistance mechanisms to second-line treatments for throat infections such as macrolides (40). Interestingly, two isolates (#28 and #30, accession numbers CP167004 and CP167002, respectively) were resistant to quinolones. Since these molecules are rarely used to treat GAS infections, this resistance potentially suggests a “bystander selection” during the treatment of other bacterial infections.

The findings of this study must be approached with some limitations. Specifically:

- the three-tier grouping of infections (mild/moderate/iGAS) diverges from the canonical dichotomous classification (iGAS/non-iGAS) and implies the possibility of a wider degree of misclassification (e.g., genital samples are defined as mild, while they may be sampled from cellulitis). However, our conclusions were still supported when combining mild and moderate results as non-iGAS, proving to be robust;
- sequenced isolates are a representative subset when chosen considering patient age, sex, and severity. However, on account of the small number of GAS infection cases in the pre-pandemic era, practical constraints in the pandemic era, and a lag in the detection and sequencing of GAS cases in the post-pandemic era, no pre-2023 isolates were sequenced;
- only demographic data were included in this study, overlooking aspects such as host immunity (41) and co-infections (42).

Additionally, while iGAS were unlikely to have been missed, milder cases may have been underreported during the pandemic due to reduced healthcare visits. Genomic data are increasing and will be one of the most relevant resources necessary to face the current GAS upsurge for the development of population-genomics-informed vaccines (43).

Combining clinical microbiology, epidemiological analyses, and genomics, this study confirms the recent upsurge of *S. pyogenes* and tries to untangle the factors underlying this phenomenon, which can be roughly divided into three categories: (i) host-associated (e.g., age and sex); (ii) pathogen-associated (e.g., *emm*-type, antimicrobial resistance, and virulence factors); (iii) external (e.g., the aftermaths of the COVID-19 pandemic public health measures and/or seasonal variations).

Aside from the rise of the M1_UK_ lineage, other factors certainly deserve further attention and need to be addressed by future studies, calling our attention to the unmet need for a European and/or global GAS network.

## Data Availability

BioProject has been released at DDBJ/ENA/GenBank PRJNA1070447. Accession numbers for single isolates can be found in Table 1.

## SUPPLEMENTAL MATERIAL

Dataset S1 spectrum.02494-24-s0001.xlsx Information on laboratory-identified *Group A Streptococcus* (GAS) cases between June 2018 and June 2024.

Dataset S2 spectrum.02494-24-s0002.xlsx Single Nucleotide Polymorphisms distance matrix based on the core genes shared by the ST28 isolates sequenced in this study.

Dataset S3 spectrum.02494-24-s0003.xlsx Mobilome composition of the 34 GAS isolates subjected to Whole Genome Sequencing.

Table S1 spectrum.02494-24-s0004.xlsx Phenotype, Integrative Conjugative Element (ICE) and resistance gene content of the 34 *Group A Streptococcus* (GAS) isolates subjected to Whole Genome Sequencing.

ASM does not own the copyrights to Supplemental Material that may be linked to, or accessed through, an article. The authors have granted ASM a non-exclusive, world-wide license to publish the Supplemental Material files. Please contact the corresponding author directly for reuse.

## References

[1] Efstratiou A, Lamagni T. n.d. Epidemiology of Streptococcus pyogenes. in Streptococcus pyogenes: Basic Biology to Clinical Manifestations (eds

[2] Stevens DL, Tanner MH, Winship J, Swarts R, Ries KM, Schlievert PM, Kaplan E. 1989. Severe group a streptococcal infections associated with a toxic shock-like syndrome and scarlet fever toxin a. N Engl J Med 321:1–7. doi:10.1056/NEJM198907063210101 2659990

[3] Lynskey NN, Jauneikaite E, Li HK, Zhi X, Turner CE, Mosavie M, Pearson M, Asai M, Lobkowicz L, Chow JY, Parkhill J, Lamagni T, Chalker VJ, Sriskandan S. 2019. Emergence of dominant toxigenic M1T1 Streptococcus pyogenes clone during increased scarlet fever activity in England: a population-based molecular epidemiological study. Lancet Infect Dis 19:1209–1218. doi:10.1016/S1473-3099(19)30446-3 31519541 PMC6838661

[4] Johannesen TB, Munkstrup C, Edslev SM, Baig S, Nielsen S, Funk T, Kristensen DK, Jacobsen LH, Ravn SF, Bindslev N, et al.. 2023. Increase in invasive group a streptococcal infections and emergence of novel, rapidly expanding sub-lineage of the virulent Streptococcus pyogenes M1 clone, Denmark, 2023. Euro Surveill 28:2300291. doi:10.2807/1560-7917.ES.2023.28.26.2300291 37382884 PMC10311951

[5] Lassoued Y, Assad Z, Ouldali N, Caseris M, Mariani P, Birgy A, Bonacorsi S, Bidet P, Faye A. 2023. Unexpected increase in invasive group a Streptococcal Infections in children after respiratory viruses outbreak in France: a 15-year time-series analysis. Open Forum Infect Dis 10:ofad188. doi:10.1093/ofid/ofad188 37180594 PMC10167988

[6] Wolters M, Berinson B, Degel-Brossmann N, Hoffmann A, Bluszis R, Aepfelbacher M, Rohde H, Christner M. 2024. Population of invasive group a streptococci isolates from a German tertiary care center is dominated by the hypertoxigenic virulent M1_UK_ genotype. Infection 52:667–671. doi:10.1007/s15010-023-02137-1 38064158 PMC10954911

[7] van der Putten BCL, Vlaminckx BJM, de Gier B, Freudenburg-de Graaf W, van Sorge NM. 2023. Group a streptococcal meningitis with the M1_UK_ variant in the Netherlands. JAMA 329:1791–1792. doi:10.1001/jama.2023.5927 37027150 PMC10082416

[8] Cobo-Vázquez E, Aguilera-Alonso D, Carrasco-Colom J, Calvo C, Saavedra-Lozano J, PedGAS-net Working Group. 2023. Increasing incidence and severity of invasive group a streptococcal disease in Spanish children in 2019-2022. Lancet Reg Health Eur 27:100597. doi:10.1016/j.lanepe.2023.100597 36895202 PMC9989682

[9] Messacar K, Baker RE, Park SW, Nguyen-Tran H, Cataldi JR, Grenfell B. 2022. Preparing for uncertainty: endemic paediatric viral illnesses after COVID-19 pandemic disruption. The Lancet 400:1663–1665. doi:10.1016/S0140-6736(22)01277-6 PMC9282759 35843260

[10] Davies PJB, Russell CD, Morgan A-R, Taori SK, Lindsay D, Ure R, Brown D, Smith A. 2023. Increase of severe pulmonary infections in adults caused by M1_UK_ Streptococcus pyogenes, Central Scotland, UK. Emerg Infect Dis 29:1638–1642. doi:10.3201/eid2908.230569 37343545 PMC10370863

[11] Rodriguez-Ruiz JP, Lin Q, Lammens C, Smeesters PR, van Kleef-van Koeveringe S, Matheeussen V, Malhotra-Kumar S. 2023. Increase in bloodstream infections caused by emm1 group a Streptococcus correlates with emergence of toxigenic M1_UK_, Belgium, May 2022 to August 2023. Euro Surveill 28:2300422. doi:10.2807/1560-7917.ES.2023.28.36.2300422 37676145 PMC10486196

[12] Li Y, Rivers J, Mathis S, Li Z, Chochua S, Metcalf BJ, Beall B, Onukwube J, Gregory CJ, McGee L, et al.. 2023. Expansion of invasive group a Streptococcus M1_UK_ lineage in active bacterial core surveillance, United States, 2019‒2021. Emerg Infect Dis 29:2116–2120. doi:10.3201/eid2910.230675 37640370 PMC10521608

[13] Rümke LW, de Gier B, Vestjens SMT, van der Ende A, van Sorge NM, Vlaminckx BJM, Witteveen S, van Santen M, Schouls LM, Kuijper EJ. 2020. Dominance of M1_UK_ clade among dutch M1 Streptococcus pyogenes. Lancet Infect Dis 20:539–540. doi:10.1016/S1473-3099(20)30278-4 32359464

[14] Gouveia C, Bajanca-Lavado MP, Mamede R, Araújo Carvalho A, Rodrigues F, Melo-Cristino J, Ramirez M, Friães A. 2023. Sustained increase of paediatric invasive Streptococcus pyogenes infections dominated by M1_UK_ and diverse emm12 isolates, Portugal, September 2022 to May 2023. Euro Surveill 28:2300427. doi:10.2807/1560-7917.ES.2023.28.36.2300427 37676143 PMC10486195

[15] van der Putten BCL, Bril-Keijzers WCM, Rumke LW, Vestjens SMT, Koster LAM, Willemsen M, van Houten MA, Rots NY, Vlaminckx BJM, de Gier B, van Sorge NM. 2023. Novel emm4 lineage associated with an upsurge in invasive group A streptococcal disease in the Netherlands, 2022. Microb Genom 9:001026. doi:10.1099/mgen.0.001026 PMC10327499 37261428

[16] Stevens DL, Bryant AE. 2022. *Streptococcus pyogenes* impetigo, erysipelas, and cellulitis. In Ferretti JJ, Stevens DL, Fischetti VA (ed), Streptococcus pyogenes: Basic Biology to Clinical Manifestations. University of Oklahoma Health Sciences Center, Oklahoma City (OK). 36479753

[17] Stevens DL, Bryant AE. 2022. Severe *Streptococcus pyogenes* infections. In Ferretti JJ, Stevens DL, Fischetti VA (ed), Streptococcus pyogenes: Basic Biology to Clinical Manifestations. University of Oklahoma Health Sciences Center, Oklahoma City (OK).

[18] Novazzi F, Colombini L, Perniciaro S, Genoni A, Agosti M, Santoro F, Mancini N. 2024. A family cluster of Streptococcus pyogenes associated with a fatal early-onset neonatal sepsis. Clin Microbiol Infect 30:830–832. doi:10.1016/j.cmi.2024.02.015 38432434

[19] Pinzauti D, Iannelli F, Pozzi G, Santoro F. 2022. DNA isolation methods for nanopore sequencing of the Streptococcus mitis genome. Microb Genom 8:000764. doi:10.1099/mgen.0.000764 35171093 PMC8942023

[20] Colombini L, Cuppone AM, Tirziu M, Lazzeri E, Pozzi G, Santoro F, Iannelli F. 2023. The mobilome-enriched genome of the competence-deficient Streptococcus pneumoniae BM6001, the original host of integrative conjugative element Tn5253, Is phylogenetically distinct from historical pneumococcal genomes. Microorganisms 11:1646. doi:10.3390/microorganisms11071646 37512819 PMC10383233

[21] Tatusova T, DiCuccio M, Badretdin A, Chetvernin V, Nawrocki EP, Zaslavsky L, Lomsadze A, Pruitt KD, Borodovsky M, Ostell J. 2016. NCBI prokaryotic genome annotation pipeline. Nucleic Acids Res 44:6614–6624. doi:10.1093/nar/gkw569 27342282 PMC5001611

[22] Bortolaia V, Kaas RS, Ruppe E, Roberts MC, Schwarz S, Cattoir V, Philippon A, Allesoe RL, Rebelo AR, Florensa AF, et al.. 2020. ResFinder 4.0 for predictions of phenotypes from genotypes. J Antimicrob Chemother 75:3491–3500. doi:10.1093/jac/dkaa345 32780112 PMC7662176

[23] De Giorgi S, Ricci S, Colombini L, Pinzauti D, Santoro F, Iannelli F, Cresti S, Piomboni P, De Leo V, Pozzi G. 2022. Genome sequence typing and antimicrobial susceptibility testing of infertility-associated Enterococcus faecalis reveals clonality of aminoglycoside-resistant strains. J Glob Antimicrob Resist 29:194–196. doi:10.1016/j.jgar.2022.03.017 35351675

[24] Patil I. 2021. Visualizations with statistical details: the “ggstatsplot” approach. JOSS 6:3167. doi:10.21105/joss.03167

[25] Wickham H. 2016. Ggplot2. Springer International Publishing, Cham.

[26] Wickham H, Averick M, Bryan J, Chang W, McGowan L, François R, Grolemund G, Hayes A, Henry L, Hester J, Kuhn M, Pedersen T, Miller E, Bache S, Müller K, Ooms J, Robinson D, Seidel D, Spinu V, Takahashi K, Vaughan D, Wilke C, Woo K, Yutani H. 2019. Welcome to the Tidyverse. JOSS 4:1686. doi:10.21105/joss.01686

[27] Li HK, Zhi X, Vieira A, Whitwell HJ, Schricker A, Jauneikaite E, Li H, Yosef A, Andrew I, Game L, Turner CE, Lamagni T, Coelho J, Sriskandan S. 2023. Characterization of emergent toxigenic M1UK Streptococcus pyogenes and associated sublineages. Microb Genom 9:000994. doi:10.1099/mgen.0.000994 PMC10210942 37093716

[28] Friães A, Machado MP, Pato C, Carriço J, Melo-Cristino J, Ramirez M. 2015. Emergence of the same successful clade among distinct populations of emm89 Streptococcus pyogenes in multiple geographic regions. MBio 6:e01780-15. doi:10.1128/mBio.01780-15 26628724 PMC4669383

[29] Flores AR, Jewell BE, Fittipaldi N, Beres SB, Musser JM. 2012. Human disease isolates of serotype M4 and M22 group a streptococcus lack genes required for hyaluronic acid capsule biosynthesis. MBio 3:e00413-12. doi:10.1128/mBio.00413-12 23131832 PMC3487777

[30] Flores AR, Chase McNeil J, Shah B, Van Beneden C, Shelburne SA. 2019. Capsule-negative emm types are an increasing cause of pediatric group a streptococcal infections at a large pediatric hospital in texas. J Pediatric Infect Dis Soc 8:244–250. doi:10.1093/jpids/piy053 30085121 PMC8938855

[31] Brenciani A, Tiberi E, Bacciaglia A, Petrelli D, Varaldo PE, Giovanetti E. 2011. Two distinct genetic elements are responsible for erm(TR)-mediated erythromycin resistance in tetracycline-susceptible and tetracycline-resistant strains of Streptococcus pyogenes. Antimicrob Agents Chemother 55:2106–2112. doi:10.1128/AAC.01378-10 21343455 PMC3088260

[32] Cochetti I, Tili E, Vecchi M, Manzin A, Mingoia M, Varaldo PE, Montanari MP. 2007. New Tn916-related elements causing erm(B)-mediated erythromycin resistance in tetracycline-susceptible pneumococci. J Antimicrob Chemother 60:127–131. doi:10.1093/jac/dkm120 17483548

[33] Lamagni T, Guy R, Chand M, Henderson KL, Chalker V, Lewis J, Saliba V, Elliot AJ, Smith GE, Rushton S, Sheridan EA, Ramsay M, Johnson AP. 2018. Resurgence of scarlet fever in England, 2014-16: a population-based surveillance study. Lancet Infect Dis 18:180–187. doi:10.1016/S1473-3099(17)30693-X 29191628

[34] Rivano F, Votto M, Caimmi S, Cambieri P, Castagnoli R, Corbella M, De Amici M, De Filippo M, Landi E, Piralla A, Taietti I, Baldanti F, Licari A, Marseglia GL. 2024. Invasive streptococcal Infection in children: an Italian case series. Children (Basel) 11:614. doi:10.3390/children11060614 38929194 PMC11201606

[35] Mangioni D, Fox V, Saltini P, Lombardi A, Bussini L, Carella F, Cariani L, Comelli A, Matinato C, Muscatello A, Teri A, Terranova L, Cento V, Carloni S, Bartoletti M, Alteri C, Bandera A. 2024. Increase in invasive group a streptococcal infections in Milan, Italy: a genomic and clinical characterization. Front Microbiol 14:1287522. doi:10.3389/fmicb.2023.1287522 38274761 PMC10808429

[36] Smith AP, Williams EP, Plunkett TR, Selvaraj M, Lane LC, Zalduondo L, Xue Y, Vogel P, Channappanavar R, Jonsson CB, Smith AM. 2022. Time-dependent increase in susceptibility and severity of secondary bacterial infections during SARS-CoV-2. Front Immunol 13:894534. doi:10.3389/fimmu.2022.894534 35634338 PMC9134015

[37] Kennis M, Tagawa A, Kung VM, Montalbano G, Narvaez I, Franco-Paredes C, Vargas Barahona L, Madinger N, Shapiro L, Chastain DB, Henao-Martínez AF. 2022. Seasonal variations and risk factors of Streptococcus pyogenes infection: a multicenter research network study. Ther Adv Infect Dis 9:20499361221132101. doi:10.1177/20499361221132101 36277299 PMC9585558

[38] Lamagni TL, Darenberg J, Luca-Harari B, Siljander T, Efstratiou A, Henriques-Normark B, Vuopio-Varkila J, Bouvet A, Creti R, Ekelund K, Koliou M, Reinert RR, Stathi A, Strakova L, Ungureanu V, Schalén C, Jasir A. 2008. Epidemiology of severe Streptococcus pyogenes disease in Europe. J Clin Microbiol 46:2359–2367. doi:10.1128/JCM.00422-08 18463210 PMC2446932

[39] Nelson GE, Pondo T, Toews K-A, Farley MM, Lindegren ML, Lynfield R, Aragon D, Zansky SM, Watt JP, Cieslak PR, Angeles K, Harrison LH, Petit S, Beall B, Van Beneden CA. 2016. Epidemiology of invasive group a streptococcal infections in the United States, 2005-2012. Clin Infect Dis 63:478–486. doi:10.1093/cid/ciw248 27105747 PMC5776658

[40] Yoon YK, Park CS, Kim JW, Hwang K, Lee SY, Kim TH, Park DY, Kim HJ, Kim DY, Lee HJ, Shin HY, You YK, Park DA, Kim SW. 2017. Guidelines for the antibiotic use in Adults with acute upper respiratory tract infections. Infect Chemother 49:326–352. doi:10.3947/ic.2017.49.4.326 29299900 PMC5754344

[41] Brouwer S, Rivera-Hernandez T, Curren BF, Harbison-Price N, De Oliveira DMP, Jespersen MG, Davies MR, Walker MJ. 2023. Pathogenesis, epidemiology and control of group a streptococcus infection. Nat Rev Microbiol 21:431–447. doi:10.1038/s41579-023-00865-7 36894668 PMC9998027

[42] Herrera AL, Potts R, Huber VC, Chaussee MS. 2023. Influenza enhances host susceptibility to non-pulmonary invasive Streptococcus pyogenes infections. Virulence 14:2265063. doi:10.1080/21505594.2023.2265063 37772916 PMC10566429

[43] Davies MR, McIntyre L, Mutreja A, Lacey JA, Lees JA, Towers RJ, Duchêne S, Smeesters PR, Frost HR, Price DJ, et al.. 2019. Atlas of group a streptococcal vaccine candidates compiled using large-scale comparative genomics. Nat Genet 51:1035–1043. doi:10.1038/s41588-019-0417-8 31133745 PMC6650292

